# Brazilian Protocol for Sexually Transmitted Infections 2020: viral hepatitis

**DOI:** 10.1590/0037-8682-834-2020

**Published:** 2021-05-17

**Authors:** Geraldo Duarte, Paula Pezzuto, Tiago Dahrug Barros, Gláucio Mosimann, Flor Ernestina Martinez-Espinosa

**Affiliations:** 1 Universidade de São Paulo, Faculdade de Medicina de Ribeirão Preto, Ribeirão Preto, SP, Brasil.; 2 Ministério da Saúde, Secretaria de Vigilância em Saúde, Brasília, DF, Brasil.; 3 Fundação Oswaldo Cruz, Instituto Leônidas e Maria Deane, Manaus, AM, Brasil.

**Keywords:** Hepatitis A, Hepatitis B, Hepatitis C, Vaccines, Therapeutics, Disease prevention

## Abstract

This article discusses viral hepatitis, a theme addressed by the Clinical Protocol and Therapeutic Guidelines to Comprehensive Care for People with Sexually Transmitted Infections and, more precisely, by the Clinical Protocols and Therapeutic Guidelines for Hepatitis B and Hepatitis C and Coinfections, published by the Brazilian Ministry of Health. Besides the broad spectrum of health impairment, hepatitis A, B, and C viruses also present different transmission forms, whether parenteral, sexual, vertical, or fecal-oral. Among the strategies suggested for the control of viral hepatitis, in addition to behavioral measures, are expanded diagnosis, early vaccination against hepatitis A and hepatitis B viruses, and access to available therapeutic resources. Considering vertical transmission of the hepatitis B and hepatitis C viruses, screening for pregnant women with chronic hepatitis B and C is an essential perinatal health strategy, indicating with precision those who can benefit from the prophylactic interventions.

## Highlighted excerpt

Viral hepatitis A, B, and C are responsible for more than 1.34 million deaths worldwide every year, from which 66% are the result of hepatitis B, 30% of hepatitis C, and 4% of hepatitis A.

## FOREWORD

Viral hepatitis is a topic present in the Clinical Protocol and Therapeutic Guidelines (CPTG) for Comprehensive Care for People with Sexually Transmitted Infections (STI). It is more specifically approached in the CPTG for hepatitis B and Coinfections and the CPTG for Hepatitis C and Coinfections, both published by the Health Surveillance Department of the Brazilian Ministry of Health. The National Committee approved such documents for the Incorporation of Technologies in the Brazilian National Health System (Conitec)^1^ ^-^ ^3^. For this article's elaboration, there were a selection and analysis of available evidences from literature and discussion with specialists in STI in 2020. 

## EPIDEMIOLOGICAL ASPECTS

Viral hepatitis A, B, and C are caused by viruses presenting primary hepatic tissue tropism, posing a significant challenge to public health worldwide. Such infections are responsible for more than 1.34 million deaths worldwide every year, from which 66% are the result of hepatitis B, 30% of hepatitis C, and 4% of hepatitis A^4^. These deaths mainly arise from complications of hepatitis chronic forms, such as hepatic failure, cirrhosis, and hepatocellular carcinoma^5^. Such significant data made the World Health Organization (WHO) assume, as one of its goals, ending viral hepatitis until 2030^6^. 

From 1999 to 2019, the Notifiable Disease Information System registered 673,389 viral hepatitis cases in Brazil. From such amount, 168,036 (25%) were hepatitis A, 27,890 (36.8%), hepatitis V, 253,307 (37.6%), hepatitis C, and 4,156 (0.6%), hepatitis D^7^. 

Hepatitis A virus (HAV) belongs to the *Picornaviridae* family, and its genome consists of ribonucleic acid (RNA), commonly transmitted through the fecal-oral route, contaminated food, and water ingestion. Its sexual transmission has been specially reported in men who have sex with men, which reinforces the need for including prevention measures among adults^8^ ^-^ ^12^. 

Hepatitis B virus (HBV) belongs to the *Hepadnaviridae* family, whose genetic variations lead to ten different genotypes. The genotypes have relevant epidemiologic, clinical, and therapeutical aspects^13^ ^,^ ^14^. Among the viruses considered hepatotropic, it is the only one with genome material composed of deoxyribonucleic acid (DNA). The most frequent HBV transmission modes are parenteral or percutaneous exposure, either vertical or sexual^7^. Blood is the most important transmission vehicle, but other fluids can transmit HBV as well, such as semen and vaginal content^15^. Predominant transmission routes vary according to HBV infection prevalence. In areas with high prevalence, perinatal and children's close contact routes are the virus's primary transmission forms^16^ ^,^ ^17^. In low-prevalence areas, percutaneous route and sexual contact are the contamination forms posing the greatest risk^18^. 

Hepatitis C virus (HCV) belongs to the *Flaviviridae* family, whose genetic material is composed of a positive-sense single strand RNA, and its genetic variation allows recognizing seven different genotypes^19^. Its transmission takes place through percutaneous, sexual, and vertical exposure. The occurrence frequency for each of such categories varies according to the studied population and associated factors concomitance. However, it should be highlighted that the parenteral route is more efficient and prevalent in HCV transmission than sexual and vertical transmission. The highest number of new infections has been observed among injectable drug users and through syringe and needle sharing^20^ ^,^ ^21^. 

It is known that HCV sexual transmission is more frequent among men who have sex with men^22^. As for other sexually transmitted infections, the presence of other STI, ulcerative or not, for example, human immunodeficiency virus (HIV), and unprotected sexual practices, especially those posing the greater risk of mucous bleeding (anal sex without lubricant, upper limb introduction in the vagina or anus, group sex, sexual object sharing, and sex under psychoactive drug effects), are a significant group of situations and factors facilitating HCV transmission^23^ ^,^ ^24^. Although HCV transmission is lower in people with heterosexual habits, and it is higher among those with a large number of sex partners or who perform anal sex^21^. 

## CLINICAL ASPECTS

Hepatitis A is self-limited, it does not evolve to a chronic disease, and its main control form is the vaccine^25^. Its incubation period varies from 15 to 50 days, and it is symptomatic in 70% of adults^26^ ^,^ ^27^. It is characterized by a sudden start of nausea, vomit, anorexia, fever, discomfort, and abdominal pain, followed by jaundice, choluria, acholia, and pruritus^28^. People with HAV infection transmit the virus during the incubation period, which lasts from one to six months, persisting up to one week after jaundice starting^29^. 

HBV hepatitis can be a chronic or acute disease. In the acute form, around 70% of the cases present the subclinical form, and 30% the jaundice form, which is linked to a possibly more severe disease course^30^ ^,^ ^31^. In the acute phase, clinical manifestations, such as anorexia, weakness, discomfort, nausea, jaundice, choluria, and abdomen right upper quadrant pain cannot be distinguished from other viral hepatitis^32^.

In HBV hepatitis, the progression rate of acute to chronic infection in immunocompetent individuals is determined mainly by the age when the infection takes place, being 90% in vertical transmission^33^, 20% to 50% from one to five years old^34^ ^,^ ^35^, and 0% to 10% after adolescence^12^ ^,^ ^36^. In its chronic form, hepatitis B is frequently asymptomatic, but it can evolve to chronic hepatic failure, cirrhosis, and hepatocellular carcinoma^37^. 

HCV infection has a high chronification rate, and around 50% to 85% of people evolve to the chronic infection form. Symptoms are present in a minority of cases (20 to 30%), and are mainly unspecific manifestations such as tiredness, changes in sleeping, nausea, diarrhea, abdominal pain, anorexia, myalgia, arthralgia, weakness, behavior changes, and weight loss^38^ ^,^ ^39^. Extrahepatic manifestations include cryoglobulinemia, membranoproliferative glomerulonephritis, autoimmune thyroiditis, porphyria cutanea tarda, among others^40^. In case of lack of spontaneous viral elimination or treatment, an average of 20% of HCV infection cases evolve to cirrhosis over time^41^. 

## DIAGNOSIS

Hepatitis A, B, and C diagnosis is based on detecting serological (specific viral antigens and antibodies) and molecular (viral nucleic acid) markers in the infected person's blood, serum, plasma, or oral fluid, through immunoassays or molecular biology techniques^12^ ^,^ ^42^ ^,^ ^43^. Rapid test incorporation to the Brazilian National Health System (SUS) expended testing and early detection opportunities of such infections. They can be conducted in places without laboratory infrastructure or hard to access^44^. 

For acute hepatitis A diagnosis, immunoassay tests detecting anti-HAV IgM antibodies in serum (up to six months after symptom starting) are used. IgG anti-HAV antibodies search, either through anti-HAV IgG or total anti-HAV (IgM and IgG), helps identify non-immunized or previously infected individuals. Such examinations must be requested for people exposed to this infection risk situation^44^ ^,^ ^45^. Anti-HAV IgG antibodies presence indicates long-lasting immunity^46^. 

**FIGURE 1:**
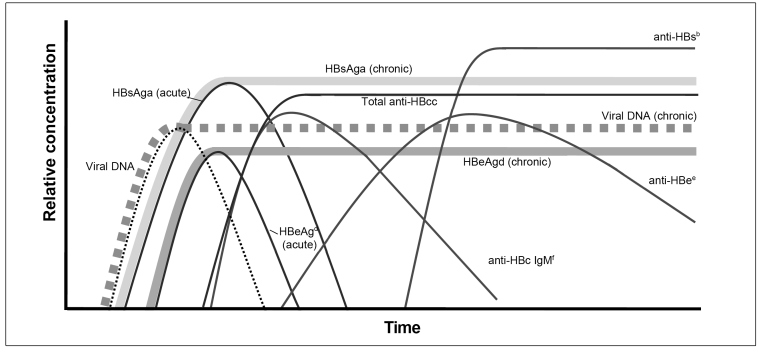
Hepatitis B virus infection serological markers according to the infection evolution time.

**Source:** altered and adapted from Sablon & Shapiro, 2005^47^.

**Notes:** a) HBsAg - hepatitis B virus surface antigen (HBV); b) Anti-HBs - antibody against HBV surface antigen; c) Total Anti-HBc - IgM and IgG class antibody against HBV core antigen; d) HBeAg - HBV antigen “e”; e) Anti-HBe - antibody against HBV antigen "e"; f) Anti-HBc IgM - IgM class antibody against HBV core antigen.

**FIGURE 2:**
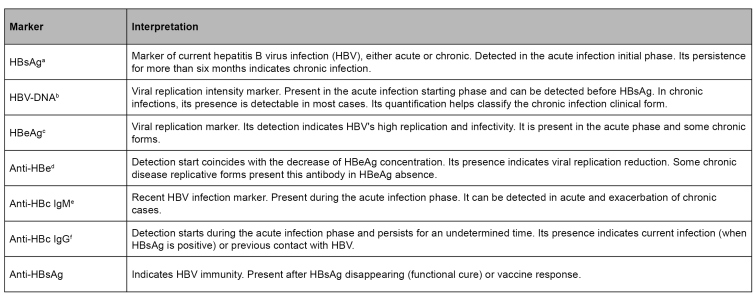
Hepatitis B virus infection markers interpretation.

**Source:** adapted from Liang, 2009^48^.

**Notes:** a) HBsAg - HBV surface antigen; b) HBV-DNA - viral nucleic acid; c) HBeAg - HBV antigen “e”; d) Anti-HBe - antibody against HBV antigen "e"; e) Anti-HBc IgM - IgM class antibody against HBV core antigen; f) Anti-HBc IgG - IgC class antibody against HBV core antigen; g) Anti-HBs - antibody against HBV surface antigen.

Most of the people with HBV infections are asymptomatic and diagnosed in the chronic disease phase. Figure 1 shows the variation time dynamics of infection markers^47^. The succinct definition of such markers is given in Figure 2 ^48^. For infection screening, an immunoassay laboratory test or a rapid test is used for detecting the HBV surface antigen (HBsAg). If positive, the diagnosis is supplemented by total anti-HBc, and, if possible, molecular test (HBV-DNA). HBeAg, anti-HBe, and anti-HBs, alongside the other markers, help in the clinical phase assessment and the infection evolution monitoring^16^ ^,^ ^37^ ^,^ ^42^ ^-^ ^44^ ^,^ ^49^. 

In acute hepatitis B, HBsAg, HBeAg, anti-HBc IgM, and HBV-DNA are the first markers to be detected. HBsAg presence confirms the infection, and it can be detected from two to 12 weeks after exposure to the virus. Its disappearance indicates that the infection has been resolved and takes place weeks after the detection, lasting for up to six months. Anti-HBc IgM disappears after the acute infection control but can be detected during flares of chronic hepatitis B^48^. 

HBeAg marks intense viral replication, and its detection is associated with high HBV infectiousness^48^. Anti-HBc IgG antibodies also have an early appearance, and they usually persist for life. They cannot classify clinical form because all infected individuals, either cured or not, present this marker. It has an epidemiological use because it indicates the previous contact with the virus or current infection. Anti-HBe presence indicates recovery starting, but many individuals presenting the disease chronic replicative form can have this marker in case of HBeAg absence^50^. Anti-HBs marker arises during the convalescent phase, after HBsAg disappearing. Its presence indicates immunity against HBV. In case of lack of anti-HB, the total anti-HBc is the only isolated marker of the previous infection. In individuals not exposed to HBV (negative anti-HBc IgG), anti-HBs antibodies presence indicate immunity through vaccine response for hepatitis B^51^. 

If patients do not achieve acute infection resolution, some individuals become HBV chronic carriers. In such cases, HBsAg persistence for more than six months indicates chronic infection. In chronic hepatitis B, HBsAg and HBV-DNA markers stay present and detectable. The HBeAg and anti-HBe assessment, together with HBV-DNA and ALT, help monitor and assess the clinical infection phase. 

Hepatitis B testing can be offered or requested for every person with high vulnerability to the disease. Those whose results are negative must be vaccinated^16^ ^,^ ^44^. 

In HCV infection, most infected people are asymptomatic, and tracking is conducted through anti-HCV antibody detection using immunoassay tests or rapid tests. In people with reactive results, the diagnosis is supplemented by a molecular test, in this case, the reverse transcriptase-polymerase chain reaction (RT-PCR) for detecting HCV's RNA, confirming active, acute, or chronic infection. Hepatitis C testing must be requested for all individuals in risk situations regarding this infection^12^ ^,^ ^52^.

## TREATMENT

A hepatitis A-specific antiviral treatment is not available. There are only medications for relieving symptoms, which generally disappear in two months^53^. 

Only support measures are enough during HBV acute infection, considering more than 90% present spontaneous resolution. In case of need for treatment, reverse transcriptase inhibitors are used^54^. In Brazil, the choice is tenofovir disoproxil fumarate or entecavir^37^. Preventive measures must be taken for all exposed contacts, indicating immunoglobulin and vaccination for serum-negative cases or those presenting unknown serology, according to the set criteria^55^. 

After six months of HBsAg persistence in blood, HBV infection is considered chronic, and it must be clinically and virologically assessed for a decision on the need for drug therapy. Hepatitis B chronic form treatment aims mainly at viral suppression, thus avoiding the hepatopathy's evolution and death. HBsAg disappearance and seroconversion for antiHBs (functional cure) would be the ideal result, but it is rarely achieved. When such an objective is not attained, anti-HBe emergence, viral load reduction, and hepatic enzyme normalization are alternative outcomes^37^ ^,^ ^56^. 

Due to its complexity, this treatment must be set under specialist guidance because it depends on multiple clinical and laboratory variables, such as the presence of significant hepatic disease, immune response to infection, viral load, and risk factors for disease progression (age and hepatocellular carcinoma family history)^37^ ^,^ ^55^. Liver biopsy can help assess the tissue aggression degree and cases in need of dismissing base liver disease^57^. The definition of HBV chronic infection antiviral therapy depends, at first, on the hepatopathy degree, on the concentration of aminotransferases, and viral load (HBV-DNA)^58^. 

HCV infection can be objectively cured by using direct-action antiviral treatment. However, as it is an asymptomatic infection in most cases, a significant number of infected people are not diagnosed, many patients remain without therapy^52^. This treatment must be followed by a specialist, considering its complexity.

**FIGURE 3:**
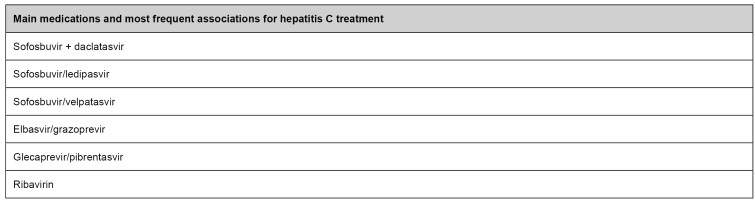
Main medications and most frequent associations for hepatitis C treatment.

**Source:** adapted from the Clinical Protocol and Treatment Guidelines for Hepatitis C and Coinfections^41^.

Therapeutic alternatives for HCV infection treatment incorporated into SUS show high therapeutic effectiveness, confirmed by a sustained virological response (Figure 3). When comparing similar clinical situations, all proposed schemes present analogous effectiveness. Characteristics differentiating them are recommendations for certain populations, ease posology, more affordable control, and cost. The therapy aims to sustain virological response, which means viral RNA's continuous disappearance after 12 to 24 weeks after treatment^41^. 

## SURVEILLANCE, PREVENTION, AND CONTROL

Viral hepatitis case notification is compulsory for all Brazilian states since 2016. All suspected cases must also be reported weekly to the health authority^59^. 

According to the Special Immunobiological Reference Center's criteria, HAV infection prevention depends on the improvement of the population's sanitary conditions and vaccination (children up to five years old and people exposed to greater risk of infection)^56^. However, in adults, the sexual transmission must also be taken into account, indicating and encouraging safe sex practices^12^. In case of hepatitis A risk exposure, the testing is recommended, and negative people can be vaccinated up to 14 days after exposure^29^. 

For HBV infection control, the best strategy is vaccination, which presents high effectiveness percentages varying according to age (preterm children and people older than 60 years show lower proportions of serological conversion) and the presence of comorbidities, such as hemodialysis, hepatopathies, cancers, and people with HIV^33^ ^,^ ^56^ ^,^ ^60^ ^-^ ^62^. 

Ninety percent of HBV infections acquired in the perinatal period evolving to the chronic form gives room for universal vaccination strategies of all newborns, regardless of the mother is a chronic virus carrier^56^ ^,^ ^60^. This strategy is crucial for achieving the goal of controlling hepatitis B up to 2030^6^ ^,^ ^63^. 

Recommendations and care with people using illicit drugs and those performing adequate unprotected sex activity are strategies that can bring better results in HCV infection reduction^6^ ^,^ ^21^ ^-^ ^24^. Another way of controlling HCV infections is the direct-action antiviral therapy, whose success extended the discussion on treatment as a disease prevention strategy^64^. 

## SPECIAL POPULATIONS AND SITUATIONS

The approach to HAV, HBV, and HCV hepatitis during pregnancy requires attention to the prophylactic aspects encompassed both in this virus horizontal and vertical transmission. For risk exposure reduction and control, primary prophylaxis sanitary and behavioral principles are needed, together with active and passive immunoprophylaxis, when avaliable^65^ ^,^ ^66^. It is known that HBV and HCV vertical transmission control depends on the universal screening of such infections during prenatal care. In general, during prenatal care, it is important to identify if non-infected pregnant women's partners carry such infections, allowing them to reduce acute infections during pregnancy, for example, including the partner in prenatal care services^67^.

### Hepatitis A during pregnancy

The short viremia period and care during delivery (avoiding the fetus' contact with the mother's stool) justify HAV vertical transmission's rare character^68^. Vaginal delivery is the recommendation for such women, and natural breastfeeding is authorized^69^. 

Both immunoglobin and the HAV vaccine are safe for being used during pregnancy. In case of exposure to clinical, occupational, and lifestyle risk factors, in addition to travels to areas with high prevalence, immunoprophylaxis can be used^12^ ^,^ ^70^. 

### Hepatitis B during pregnancy

The earlier the infection takes place, the higher HBV infection chronicity rates are^71^. For this reason, controlling vertical transmission can reduce chronic hepatitis, cirrhosis, and hepatocellular carcinoma^72^ ^,^ ^73^. 

HBsAg search is recommended as soon as possible in prenatal care, and its repetition is recommended at the moment of delivery^74^. For seronegative pregnant women, the three-dose vaccine scheme must start. The vaccine information does not exclude the need for checking if the pregnant women carry HBV in all such moments because vaccine serological conversion is not assured even after three doses of the vaccine^75^. 

With normal liver function, the HBV infection prognostic is not changed by pregnancy, and the infection does not modify the pregnancy prognosis^76^ ^,^ ^77^. In case of compromised liver, maternal and perinatal prognostics can be negatively affected^65^. 

HBV vertical transmission occurrence is influenced by the viral load, the carrier's HBeAg and anti-HBe positivity, and gestational age (infection acquired by its end increases the risk for high viral load at the moment of delivery). Without prophylactic intervention, the vertical transmission risk in chronic carriers varies from 5% to 30% (8% mean). However, if the pregnant woman carries HBeAg, the vertical transmission rate is high but extremely variable, taking place from 80% to 90% of cases^4^ ^,^ ^33^ ^,^ ^65^. 

During the prenatal care of HBV chronic carriers, all invasive conducts on the fetus and amniotic sac (amniocentesis and cordocentesis) are contraindications. When there is risk exposure, immunoprophylaxis is recommended, using, in different muscle groups, the first vaccine dose and hyperimmune hepatitis B immunoglobulin (0.06mL/kg of body weight, maximum 5mL, intramuscular)^56^ ^,^ ^74^ ^,^ ^78^. 

**FIGURE 4:**
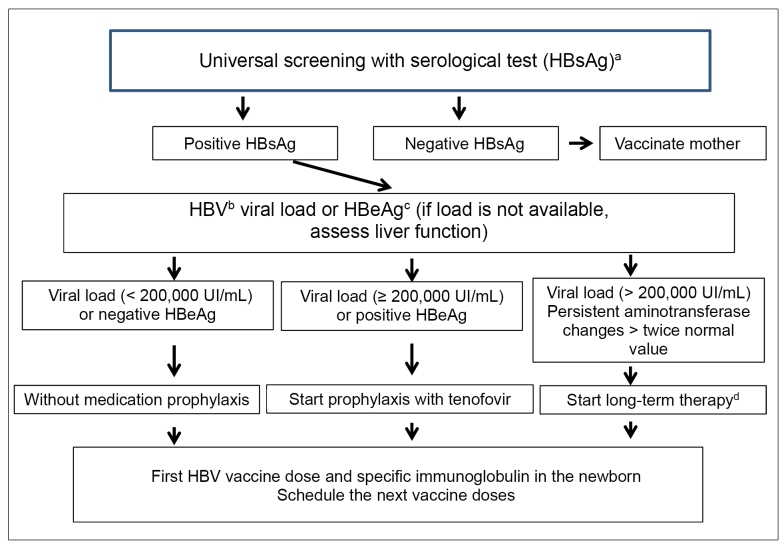
Algorithm of preventive interventions for hepatitis B virus vertical transmission prevention.

**Source:** altered and adapted from World Health Organization, 2020^71^.

**Notes:** a) HBsAg - HBV surface antigen; b) HBV: Hepatitis B virus; c) HBeAg - HBV antigen “e”; d) Start long-term therapy with tenofovir disoproxil fumarate because entecavir is not safe for breastfeeding women^80^^,^^81^.

In addition to the usual laboratory routine, the following examinations must be requested for pregnant women carrying HBV: liver function test, infection markers still not requested (HBeAg, anti-HBc, anti-HBe, anti-HBs), and viral load. All pregnant women presenting HBV viral load higher than 200,000UI/mL (corresponding to one million copies/mL) or HBeAg positivity must undergo vertical transmission prophylaxis. Changes in liver function indicate the need for long-term treatment and not only prophylaxis (Figure 4)^63^. 

For HBV vertical transmission prophylaxis, using inhibitors of the nucleoside reverse transcriptase (lamivudine, tenofovir, and telbivudine) is recommended^63^ ^,^ ^79^. In Brazil, the option is tenofovir disoproxil fumarate (300mg, *per os*, single daily dose), started from the 28^th^ week of pregnancy, lasting to postpartum period to prevent the possible increase of viral load^74^. Considering the need for long-term treatment in pregnant women carrying HBV, tenofovir disoproxil fumarate is also used. Entecavir using during pregnancy is not considered safe, and it must be avoided in such a period^80^. Likewise, its use is not recommended while breastfeeding^81^.

Vaginal delivery is authorized to women carrying HBV, preventing episiotomy and deliveries using instruments (forceps or vacuum) and rapidly clamping the umbilical cord. Natural breastfeeding is also allowed^82^. 

All newborns from women with HBV must take the vaccine first dose and HBV-specific immunoglobulin (0.5mL intramuscular), preferably in the first 12 hours of their lives, as presented in the algorithm Figure 4. After this first dose, there are many schemes for administering additional vaccine doses, some with two, others with three doses^63^. Countries like China, Malaysia, and Hong Kong, among others, use the schedule with two additional doses administered in the second and sixth months of life of the newborn^83^ ^-^ ^85^. In the United States and Canada, the schedule with two additional doses administered in the second and sixth months of life is also used. However, in preterm newborns or cases of weight lower than 2.0kg, the vaccine scheme considers three additional doses administered in the second, fourth, and sixth months of life^75^ ^,^ ^86^. In Brazil, the National Immunization Program recommends the vaccine scheme with three additional doses to the one administered at the moment of birth, using combined vaccines (pentavalent vaccine) administered in the second, fourth, and sixth months of life, regardless of newborn's prematurity or weight^74^ ^,^ ^87^. 

Whether the vaccine scheme is used, the newborn's situation must be followed-up until infection confirmation or disregarding and assessed serum conversion^71^. In Brazil, such assessment is recommended from 30 and 60 days after the last dose of anti-HBV vaccine^74^. 

### Hepatitis C during pregnancy

In 2020, the Ministry of Health added the universal screening of HCV infection during pregnancy^88^. As there is no pharmacological prophylaxis for reducing HCV vertical transmission, there is resistance to screening the virus during prenatal care^4^. However, behavioral and assistance strategies minimize such risk, such as prophylactically and therapeutically controlling other infections, forbidding invasive propaedeutics on the amniotic sac and the fetus, and preventing long-term chorioamniorrhexis. During delivery, the use of episiotomy must be avoided. The rapid clamping of the umbilical cord is recommended^65^ ^,^ ^89^ ^,^ ^90^. Also, it is one more opportunity for identifying HCV carriers who can benefit from the treatment later.

HCV vertical transmission occurs from 3.8% to 7.8% of pregnant women with chronic infection, showing a positive relationship with the viral load^91^ ^-^ ^93^. Such vertical transmission rates also vary depending on the gestational age when the acute infection occurs and on the presence of comorbidities, such as HIV infection^94^ ^,^ ^95^. There is no confirmation that HCV can cause fetal malformation^96^. 

HCV infection in pregnant women is associated with adverse perinatal prognostics, such as fetal growth restriction and prematurity^97^ ^,^ ^98^. Such results do not seem to arise only from HCV effects but from the different factors coexisting in pregnant women^94^. Regardless of the action taken alone or in association, the need to identify pregnant women with this infection and provide appropriate prenatal care and follow-up for their children is underlined^88^ ^,^ ^99^. Both vaginal delivery and natural breastfeeding are authorized for women with HCV^66^ ^,^ ^79^ provided that there is no concomitance with HIV infection^100^. 
